# Psychometric Properties of Screening Questionnaires for Children With Handwriting Issues

**DOI:** 10.3389/fpsyg.2019.02937

**Published:** 2020-01-21

**Authors:** Katarína Šafárová, Jiri Mekyska, Vojtěch Zvončák, Zoltán Galáž, Pavlína Francová, Barbora Čechová, Barbora Losenická, Zdeněk Smékal, Tomáš Urbánek, Jana Marie Havigerová, Sara Rosenblum

**Affiliations:** ^1^Department of Psychology, Masaryk University, Brno, Czechia; ^2^Department of Telecommunications, Brno University of Technology, Brno, Czechia; ^3^Department of Occupational Therapy, University of Haifa, Haifa, Israel

**Keywords:** developmental dysgraphia, reliability, validity, HPSQ, HPSQ-C

## Abstract

Dysgraphia (D) is a complex specific learning disorder with a prevalence of up to 30%, which is linked with handwriting issues. The factors recognized for assessing these issues are legibility and performance time. Two questionnaires, the Handwriting Proficiency Screening Questionnaire (HPSQ) for teachers and its modification for children (HPSQ-C), were established as quick and valid screening tools along with a third factor – emotional and physical well-being. Until now, in the Czechia, there has been no validated screening tool for D diagnosis. A study was conducted on a set of 294 children from 3rd and 4th year of primary school (132 girls/162 boys; *M*_*age*_ 8.96 ± 0.73) and 21 teachers who spent most of their time with them. Confirmatory factor analysis based on the theoretical background showed poor fit for HPSQ [χ^2^(32) = 115.07, *p* < 0.001; comparative fit index (CFI) = 0.95; Tucker–Lewis index (TLI) = 0.93; root mean square error of approximation (RMSEA) = 0.09; standard root mean square residual (SRMR) = 0.05] and excellent fit for HPSQ-C [χ^2^(32) = 31.12, *p* = 0.51; CFI = 1.0; TLI = 1.0; RMSEA = 0.0; SRMR = 0.04]. For the HPSQ-C models, there were no differences between boys and girls [Δχ^2^(7) = 12.55, *p* = 0.08]. Values of McDonalds’s ω indicate excellent (HPSQ, ω = 0.9) and acceptable (HPSQ-C, ω = 0.7) reliability. Boys were assessed as worse writers than girls based on the results of both questionnaires. The grades positively correlate with the total scores of both HPSQ (*r* = 0.54, *p* < 0.01) and HPSQ-C (*r* = 0.28, *p* < 0.01). Based on the results, for the assessment of handwriting difficulties experienced by Czech children, we recommend using the HPSQ-C questionnaire for research purposes.

## Introduction

Handwriting is a complex task requiring a perfect combination of motor and cognitive skills (Feder and Majnemer, 2007; McCutchen, 2011). During childhood, children learn to write at both qualitative as well as quantitative levels, which in general spans a period of approximately 10 years (from the age of 5 years, when a child first encounters this task, up to the age of 15 years, when a child is supposed to be comfortable with writing on a daily basis), i.e., the handwriting should meet the expectations of being legible, fast enough, etc. (Ziviani and Wallen, 2006; Accardo et al., 2013). Handwriting forms the basis of a child’s capability of being educated, the ability to express his/her ideas, and to communicate throughout his/her life (Graham, 1990). Therefore, writing issues could consistently cause problems in everyday life, they could lower self-esteem and reduce academic achievement (Blöte and Hamstra-Bletz, 1991; Dunford et al., 2005; Feder and Majnemer, 2007; Dinehart, 2015), e.g., teachers tend to give worse grades to children whose handwriting is poor (Briggs, 1980; Chase, 1986; Graham et al., 2000). Although children frequently use new technology, such as smartphones and tablets, handwriting is still an important part of their education process.

Dysgraphia (D) occurs in literature as a subtype of specific learning disorder (SpLD). It can be found in the 10th edition of International Statistical Classification of Diseases and Related Health Problems (ICD-10), a medical classification system established by the World Health Organization (WHO), in Specific developmental disorders of scholastic skills, more specifically, as a Disorder of written expression (F81.81; World Health Organization [WHO], 1992). This classification is used in the Czechia where the questionnaires were adapted. D is usually defined as a disturbance in the production of the written process. Döhla and Heim (2016) defined developmental D as a problem in the acquisition of writing skills and report that a child with D is below the expected level of writing performance in comparison with her/his peers. Children with this disorder are not identified as having neurological problems or mental retardation (Hamstra-Bletz and Blöte, 1990).

The prevalence of D ranges between 10 and 30% (Cermak and Bissell, 2014; Döhla and Heim, 2016). In the Czechia, the prevalence of SpLD is estimated to be 3–5% (Zelinková, 2003; Kejřová and Krejčová, 2015); nevertheless, there is a lack of sole statistics for D. Besides, boys are generally considered to be worse in legibility and quality of handwriting than girls (Hawke et al., 2009), which results in two to three times higher prevalence (Snowling, 2005; Katusic et al., 2009). The differences in prevalence percentage are due to different diagnosis criteria and a lack of information about D. In comparison, the keyword “dyslexia” has 12 120 search results according to the Web of Science, while “D” has only 832. If we want to provide children with D with better care, it is necessary to introduce better diagnostic and screening tools and to learn more about underlying processes and their manifestation.

Generally, two factors are used to assess and/or define poor handwriting: (1) legibility and (2) performance time (Graham et al., 1998; Koziatek and Powell, 2002; Rosenblum et al., 2003; Germano et al., 2016). For that reason, there are plenty of tests which have been designed to assess these two factors. Legibility is generally understood as an extent of readability of the text or as the ease with which the letters or words are recognized (Amundson, 1995). Rosenblum et al. (2003) distinguished between global and analytic types of tools used to assess legibility. The global scales are based on the overall judgment of the sole factor of legibility. The analytic ones focus on different aspects of handwriting (e.g., letter form, size, slant, spacing, alignment, spelling and grammatical mistakes, speed, and spatial organization). It is assumed that all the features are part of the legibility factor. Performance time, also referred to as speed, is usually measured as the number of letters or words per time unit (1–5 min). Recent reviews of these tests were conducted by several authors (Feder and Majnemer, 2003; Roston et al., 2008), with the same outcome: most of them do not have proper manuals or standardization, they have old norms, and they are problematic in terms of reliability and validity.

Moreover, since a single study does not provide enough evidence to validate a test, than a design of practically useful D diagnosis tool with appropriate psychometric properties must be based on several works. Finally, concerning the replication crisis in psychology, we could not neglect the impact of drawer effect (publishing only significant and positive results) on reported findings. To overcome some of the above-mentioned limitations, in 2008, Rosenblum (2008) introduced Handwriting Proficiency Screening Questionnaire (HPSQ) that is used to assess handwriting proficiency by teachers. Later, Rosenblum and Gafni-Lachter (2015) proposed its modification (HPSQ-C) that is used by children to assess themselves (more information about these questionnaires can be found in the section “Materials and Methods”). Since clinicians also reported fatigue or pain while writing and unwillingness to do homework in children with D (Benbow, 1995; Feder et al., 2000; Tseng and Chow, 2000), Rosenblum (2008) considered these factors as important signs of D and included a well-being factor into both questionnaires. This factor was omitted in previous tests and no data on that subject exists in the literature (Engel-Yeger et al., 2009). Author of both questionnaires reported sufficient reliability and validity.

In the Czechia, the D diagnostic process includes: (1) creation of family anamnesis based on interviews with parents and child her/himself; (2) teacher’s evaluation of a child’s performance at school, where marks and written homework are analyzed; (3) psychological examination, including assessment of intellect, working memory, and visual and spatial differentiation; (4) examination of graphomotor difficulties, motor skills, laterality, quantitative analysis of grammar mistakes in written text (dictation and transcription), and qualitative analysis of observation (pen grip, sitting position, subjective assessment of temporal, spatial, kinematic and dynamic handwriting characteristics). To assess the handwriting problems a team of experts (psychologists and a special educationist) is working together. Nevertheless, in our research, we are focusing on two types of evaluations: children and teachers. We perceive those groups are usually omitted, yet very important because they are in the front line in diagnosing D.

In Czech school practice, there is no screening tool for children or for teachers which could provide quick and efficient differentiation between children with/without handwriting problems. HPSQ and HPSQ-C could bridge this gap. They are focused on three domains of non-proficient handwriting issues, which are: (1) legibility; (2) performance time; and (3) physical and emotional well-being. Previous studies indicated that these tools could be reliable and valid for screening handwriting deficits (Rosenblum, 2008; Rosenblum and Gafni-Lachter, 2015). Moreover, its assessment could be extended by computerized analysis, which makes the overall process more objective (Mekyska et al., 2017). Nevertheless, until now there have been no norms for Czech pupils that use cursive handwriting.

To sum up, D diagnosis and rating is a complex task that nowadays relies mostly on experience of teachers, psychologists, and/or occupational therapists. There is no valid screening tool which could provide fast and reliable differentiation between dysgraphic and non-dysgraphic children in schools. Therefore, the general goal of this study is to adapt the HPSQ and HPSQ-C for Czech language and check their validity and reliability. In addition, none of the previous studies compared the results of both questionnaires. They were used as a research tool, but there is no evidence of their comparison in one context (Rosenblum, 2008; Cantero-Téllez et al., 2015). We perceive this information as missing one and this step as logical, because these questionnaires contain the same items, they are just adjusted to children or their teachers. To sum up, in the range of this study we focus on:

1. Construct validity – hypothesis: (1) Factor structure of HPSQ and HPSQ-C will correspond with its theoretical background, i.e., it should have a three-factor structure: legibility (items 1, 2, and 10), performance time (items 3, 4, and 9), and physical and emotional well-being (items 5, 6, 7, and 8) (Rosenblum, 2008; Rosenblum and Gafni-Lachter, 2015).
2. Reliability analysis – hypothesis: (2) Internal consistency (McDonald’s ω) of both questionnaires will be >0.7, which is considered as an acceptable level.
3. Discriminant validity – hypotheses: (3) HPSQ and HPSQ-C will differentiate girls and boys; (4) the higher the total scores of HPSQ and HPSQ-C, the higher the average grade will be.
4. Exploration of differences between HPSQ and HPSQ-C – hypothesis: (5) There is no significant difference between total scores of HPSQ and HPSQ-C; (6) Pearson’s correlations coefficient between the same items of each questionnaire will be positive and >0.6, which is considered as a strong relationship.

## Materials and Methods

### Study Participants

In this study, we used two sources of data, i.e., data from children and their teachers, respectively. Each group filled in a related questionnaire (see the following section about the HPSQ and HPSQ-C instruments). We enrolled 294 Czech-speaking children (132 girls/162 boys; mean age 8.96 ± 0.73, HPSQ-C: *m* = 12.86, SD = 5.68) and 21 teachers who spent most of the school-time with the enrolled children (HPSQ: *m* = 11.55, SD = 6.79), in seven Czech schools (3rd and 4th class). Related demographic data for children can be found in Table 1. Thirty-three children (12.89%) were left-handed which is in line with 10–13% prevalence previously reported (Hardyck and Petrinovich, 1977; Raymond et al., 1996). Based on reports of teachers, 28.87% of children have handwriting difficulties (cf. 37.5% in Schwellnus et al., 2012). The parents of all children enrolled in the study and the teachers signed an informed consent form. Through the whole study the Ethical Principles of Psychologists and Code of Conduct released by the American Psychological Association (2019)^1^ were followed.

**TABLE 1:** Gender distribution in both classes.

	Third class	Fourth class	Total
Girls	73 (49.7%)	59 (40.1%)	132 (44.9%)
Boys	74 (50.3%)	88 (59.9%)	162 (55.1%)
Total	147 (100%)	147 (100%)	294 (100%)

### Instruments: HPSQ and HPSQ-C

The original version of HPSQ and HPSQ-C is written in Hebrew and has been consequently translated into English (Rosenblum, 2008). Questionnaires contain the same questions which are modified for person’s evaluating bias. In HPSQ, teacher is asked about her or his student’s handwriting problems and in HPSQ-C children evaluate themselves. Both questionnaires comprise of 10 items that are grouped in three factors: legibility (items 1, 2, and 10), performance time (items 3, 4, and 9), and physical and emotional well-being (items 5, 6, 7, and 8) (Rosenblum, 2008; Rosenblum and Gafni-Lachter, 2015). An example of HPSQ legibility question is “*Is the child’s handwriting readable?*” performance time question “*Does the child often erase while writing?*,” and physical and emotional well-being question “*Does the child tire while writing?*.” Every item is scored on a 5-point Likert scale ranging from 0 (never) to 4 (always). The final score (max. 40) is computed as a sum of all items, where higher sum means poorer handwriting performance. In addition, the questionnaires record information about age, class, and average grade (Czech language, English language, Maths and Fundamentals of social and natural science).

Rosenblum (2008) reports that Cronbach’s α of the HPSQ and HPSQ-C is equal to 0.90 and 0.77, respectively, indicating high to moderate reliability (Rosenblum and Gafni-Lachter, 2015). Spanish colleagues (Cantero-Téllez et al., 2015) report internal consistency of HPSQ α = 0.78. First attempts to validate this method showed only two factors in HPSQ: (1) items 3 and 9 (performance time and well-being); (2) items 1, 2, and 10 (legibility); with 67% of the variance explained (Rosenblum, 2008). These results are similar to those reported by Cantero-Téllez et al. (2015): (1) items 1, 2, and 10 (legibility); (2) the rest of items (performance time and well-being together); with the 49% of the variance explained. Another study focused on factor analysis of HPSQ-C (Rosenblum and Gafni-Lachter, 2015) found two factors: (1) items 3 and 5–9 (performance time and well-being); (2) items 1, 2, 4, and 10 (legibility); these two factors together explain 45% of the variance. Rosenblum (2008) recommended further research.

### Procedure

#### Translation Process

In the frame of this study, we performed the forward–backward translation process, where the English version was translated into Czech language (forward translation) and back into English (backward translation). As a first step the English version of both questionnaires was translated by two experts (an educational psychologist, as well as one of the authors of the study). Both had conceptual knowledge and were familiar with terminology covered by research topic. Two independent Czech versions were created and compared with minimum discrepancies.

Afterward, a third expert, a researcher in educational and school psychology, reviewed the Czech versions of the questionnaires collaboratively with one of the original translators from the previous step. The main goal was to identify inadequate concepts. In this part, the expert suggested that items 1–3, 6, and 10 should be reversed because they were negatively formulated (e.g., “*Does the child not do his/her homework?*”). This was perceived as an issue also by other researcher, e.g., Schwellnus et al. (2012) mentioned it as one of the limitations of HPSQ. In Czech language a negation could be created by prefix added to a verb, by special pronouns or adverbs. Moreover, the negation itself could make some difficulties while being cognitively processed by primary school children (Kaup et al., 2006; Lüdtke et al., 2008). Therefore, every negatively formulated item was rewritten into a positive way (in our example “*Does the child do his/her homework?*”). These items were reverse-scored during data transcription.

In the backward translation process, the Czech version of both questionnaires were translated by another researcher, who had no knowledge about the questionnaires. The final versions of HPSQ and HPSQ-C were discussed with the author Rosenblum of the both questionnaires.

#### Data Collection and Sample Size Justification

Recruitment of participants was done via e-mails to headmasters of 176 elementary schools in Brno, the capital of the east part of the Czechia. We got replies from seven schools. Two of the schools are attended by more than 500 pupils, three of them by more than 100 pupils, and two of the schools are attended by fewer than 50 pupils. Children and teachers were enrolled from both types of schools, both from larger schools in the city and its suburbs, and from smaller ones in villages.

Data were collected based on the convenience sampling method. Because there is no established rule of thumb for the sample size determination in the confirmatory factor analysis (CFA), or just with little empirical evidence (Guadagnoli and Velicer, 1988), we followed different recommendations. Some authors estimate that a sample of 100 participants would be sufficient for a measure with three or more indicators per factor (Anderson and Gerbing, 1984; MacCallum et al., 1999). Kline (1998) regards samples between 100 and 200 participants as medium sized. There are other researchers (e.g., Su et al., 2014; Fang et al., 2015) who argue that the minimum sample should be at least 200. For more information, we also refer to Osborne and Costello (2004) or Chang et al. (2018). Based on previous estimates, which consider minimum 200 participants in the sample, we justified our sample size as sufficient.

Both questionnaires HPSQ and HPSQ-C were administered in a paper–pencil form. At the beginning of testing we explained to the participants how to fill out the questionnaires, particularly in the case of the children. HPSQ was administered individually and HPSQ-C was administered to whole classes. Children were not aware of their teachers’ evaluation.

#### Data Analysis

**FIGURE 1:**
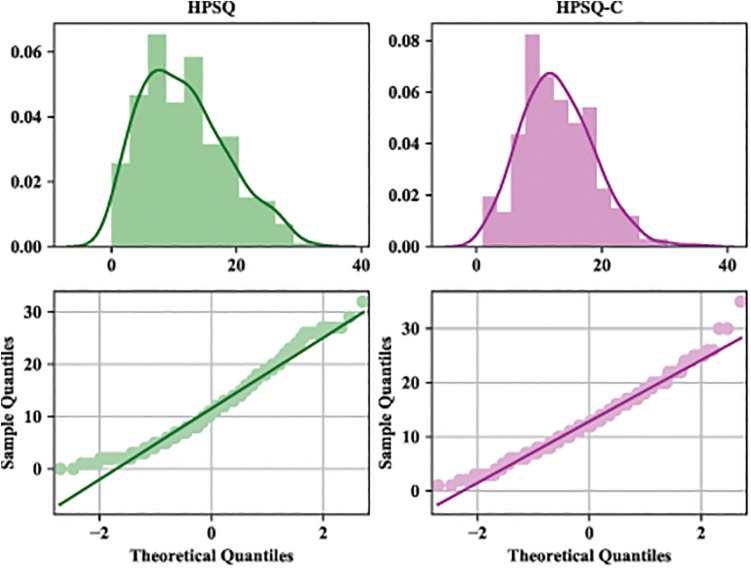
Kernel density estimation, histogram, and Q–Q plot of HPSQ/HPSQ-C total score.

Both Kolmogorov–Smirnov (*D*_294_ = 0.96, *p* < 0.001) and Shapiro–Wilk (*W*_294_ = 0.96, *p* < 0.001) tests confirmed non-normal distribution of the HPSQ total score. Same conclusions were drawn in the case of HPSQ-C (*D*_294_ = 0.98, *p* < 0.001, *W*_294_ = 0.98, *p* = 0.001). Table 2 shows the values of skewness (Sk) and kurtosis (Ku) for each item in both questionnaires. All values are in acceptable limits ± 2 (Trochim and Donnelly, 2006; Field, 2013; Gravetter and Wallnau, 2014) except for item 6 from HPSQ-C. Moreover, due to the fact that both overall distributions tend to be normal (Figure 1) and that a bigger sample size could cause that even a small deviation from normality can lead to a rejected null hypothesis of both tests (Field, 2013), in this study we decided to employ parametric tests. To analyze the data, we used IBM SPSS 25 (IBM Corp, 2017), IBM SPSS AMOS (Arbuckle, 2019), and R 3.2.2 (R Core Team, 2019).

**TABLE 2:** Skewness (Sk) and kurtosis (Ku) values of each HPSQ and HPSQ-C items.

Item		Min.	Max.	*M*	SD	Sk	SD	Ku	SD
HPSQ	1	0	3	0.95	0.89	0.53	0.14	–0.63	0.28
	2	0	3	0.83	0.85	0.73	0.14	–0.28	0.28
	3	0	4	1.08	1.07	0.69	0.14	–0.51	0.28
	4	0	4	1.94	0.92	0.17	0.14	–0.40	0.28
	5	0	4	1.38	1.02	0.30	0.14	–0.71	0.28
	6	0	3	0.38	0.71	1.94	0.14	3.33	0.28
	7	0	3	0.50	0.69	1.15	0.14	0.55	0.28
	8	0	4	1.23	0.97	0.46	0.14	–0.32	0.28
	9	0	4	2.05	1.11	0.27	0.14	–0.79	0.28
	10	0	4	1.15	0.89	0.29	0.14	–0.59	0.28
HPSQC	1	0	4	1.28	0.95	0.41	0.14	–0.18	0.28
	2	0	4	0.61	0.96	1.56	0.14	1.77	0.28
	3	0	4	0.92	1.03	0.94	0.14	0.26	0.28
	4	0	4	2.10	1.06	–0.01	0.14	–0.49	0.28
	5	0	4	1.90	1.34	0.03	0.14	–1.08	0.28
	6	0	4	0.24	0.65	3.34	0.14	12.91	0.28
	7	0	4	0.93	1.17	1.04	0.14	0.07	0.28
	8	0	4	1.60	1.36	0.33	0.14	–1.01	0.28
	9	0	4	2.32	1.15	–0.14	0.14	–0.55	0.28
	10	0	4	0.97	1.13	1.06	0.14	0.34	0.28

Construct validity was tested using the CFA to measure the fit with the theoretical background of both questionnaires. As the estimation method, we used the maximum likelihood (ML) for both questionnaires. In general, there are several indices used in the literature to check the goodness fit of CFA. For continuous data Tucker–Lewis index (TLI) and comparative fit index (CFI) should be >0.95 threshold. In addition, root mean square error of approximation (RMSEA) < 0.6 and standard root mean square residual (SRMR) < 0.08 (Hu and Bentler, 1999; Schreiber et al., 2006). For computing CFA of both questionnaires, we used the lavaan package (Rosseel, 2012) and for computing sex invariance in HPSQ-C model we used the software IBM SPSS AMOS (Arbuckle, 2019).

To assess the internal consistency of HPSQ and HPSQ-C, we calculated McDonalds’s ω, item-total correlations, and ω coefficients in the case the items are deleted. Internal consistency of the theoretical factor structure was computed using the JASP Team (2019) and of CFA model fit using R 3.2.2 software (R Core Team, 2019) with the semTools package (Jorgensen et al., 2019).

The *t*-test (sex) and Pearson’s correlation coefficient (grades) were computed for hypotheses related to the discriminant validity of HPSQ and HPSQ-C. As the last step in this article, we provide an exploration of differences and relationship between both questionnaires by computing Pearson’s correlation coefficient between items of both questionnaires and *t*-test for the differences between total scores.

## Results

### Construct Validity

#### Confirmatory Factor Analysis

The CFA was conducted with the three factors explaining the covariances of the HPSQ and HPSQ-C items separately (items 1, 2, and 10 loading on the legibility factor; items 3, 4, and 9 on the performance time factor; and items 5, 6, 7, and 8 on the physical and emotional well-being factor, which is the structure assumed by Rosenblum, 2008). All factor loadings were >0.4 and significant (*p* < 0.01) except these items: six HPSQ (0.36), three HPSQ-C (0.38) and six HPSQ-C (0.17). Parameter estimates, standardized error (SE), and standardized loadings (SLs) for both questionnaires with corresponding factors and their meanings are reported in Table 3.

**TABLE 3:** Estimates, standardized errors (SE), and standardized factor loadings (SL) from CFA for each item of HPSQ and HPSQ-C.

	Assumed factor	Item’s meaning	Item	Estimate	SE	SL
HPSQ	Legibility	Legibility	1	1.00	0.00	0.78
	Legibility	Success with reading own handwriting	2	1.00	0.05	0.78
	Legibility	Satisfaction with own handwriting	10	0.81	0.06	0.63
	Performance time	Amount of time to copying	3	1.00	0.00	0.82
	Performance time	Erasing during writing	4	0.85	0.07	0.70
	Performance time	Child frequently looks at a blackboard during copying	9	0.84	0.08	0.70
	Physical and emotional well-being	Child does not want to write	5	1.00	0.00	0.90
	Physical and emotional well-being	Doing homework	6	0.41	0.04	0.36
	Physical and emotional well-being	Child feels pain (complains)	7	0.44	0.04	0.40
	Physical and emotional well-being	Tired while writing	8	0.96	0.05	0.86
HPSQ-C	Legibility	Legibility	1	1.00	0.00	0.69
	Legibility	Success with reading own handwriting	2	0.82	0.12	0.57
	Legibility	Satisfaction with own handwriting	10	0.94	0.14	0.65
	Performance time	Amount of time to copying	3	1.00	0.00	0.38
	Performance time	Erasing during writing	4	1.55	0.37	0.59
	Performance time	Child frequently looks at a blackboard during copying	9	1.39	0.35	0.53
	Physical and emotional well-being	Child does not want to write	5	1.00	0.00	0.71
	Physical and emotional well-being	Doing homework	6	0.25	0.07	0.17
	Physical and emotional well-being	Child feels pain (complains)	7	0.71	0.14	0.50
	Physical and emotional well-being	Tired while writing	8	1.37	0.22	0.97

*All standardized loadings are statistically significant on the level *p* < 0.01.*

The global model fit of HPSQ was statistically significant [χ^2^(32) = 115.07, *p* < 0.001] with indexes values CFI = 0.95, TLI = 0.93, RMSEA = 0.09 with 90% CI (0.076, 0.113), and SRMR = 0.05. The correlations among all three latent factors were all highly statistically significant (*p* < 0.001) and positive (see in Table 4), mostly between 0.52 and 0.66 indicating that teachers who evaluated child’s issues as higher in one dimension were more likely to evaluate hers/his issues as high in the others as well. According to cut-off values mentioned in the section “Data Analysis” we do not consider these results as a good fit. The data did not support the theoretical structure.

**TABLE 4:** Latent factor correlations in HPSQ and HPSQ-C.

Questionnaire	Factor 1	Factor 2	Correlation
HPSQ	Legibility	Performance time	0.53
	Legibility	Well-being	0.53
	Performance time	Well-being	0.67
HPSQ-C	Legibility	Performance time	0.13
	Legibility	Well-being	0.23
	Performance time	Well-being	0.19

*All standardized loadings are statistically significant on the level *p* < 0.01.*

The global model fit of HPSQ-C was not statistically significant [χ^2^(32) = 31.12, *p* = 0.51] with indexes values CFI = 1.0, TLI = 1.0, RMSEA = 0.0 with 90% CI (0.000, 0.042), and SRMR = 0.04. The correlations among the three factors were all highly statistically significant and positive, but weak. The range of correlation values was from 0.13 to 0.23 (see in Table 4). It indicates that children understand those latent variables as independent. According to cut-off values mentioned in the section “Data Analysis” we consider these results as an excellent fit. The data support the theoretical structure.

In addition, we performed the CFA invariance analysis for the HPSQ-C model, where the model has two different parts for girls (*N* = 132) and boys (*N* = 162). We used the ML estimation method, where the parameters were estimated freely in each group. We tested the model on the three levels: (1) configural invariance; (2) metric invariance; and (3) scalar invariance. A non-significant result means that the model has acceptable fit when a particular level of measured invariance is assumed (Bialosiewicz et al., 2013; Chakraborty, 2017).

Requirements of configural invariance are fulfilled when the basic factor structure is invariant for both groups (Chakraborty, 2017). The model fit for girls is not excellent, but acceptable [χ^2^(32) = 37.38, *p* = 0.24] with indexes CFI = 0.96, TLI = 0.94, and RMSEA = 0.04 [90% CI (0.00, 0.08)]. The model fit for boys is acceptable [χ^2^(32) = 37.79, *p* = 0.22] with indexes CFI = 0.97, TLI = 0.96, and RMSEA = 0.03 [90% CI (0.00, 0.07)]. The global model fit is acceptable and the obtained data for unconstrained factor structure fit well with the theoretical factor structure (see indexes in Table 5). Results indicate that there are no statistically significant differences between girls’ and boys’ models [Δχ^2^(7) = 12.55, *p* = 0.08, ΔTLI = −0.004]. Based on this results we can conclude that girls and boys conceptualized the factors in same way.

**TABLE 5:** Goodness of fit measures for different factor structure for boys and girls of HPSQ-C.

Level of factor structure/measure	χ^2^	DF	*p*	CMIN/DF	RMSEA	TLI	CFI
Threshold			>0.05	<3	<0.06	>0.95	>0.95
Configural	75.31	65	0.18	1.16	0.02	0.96	0.97
Metric	88.14	72	0.10	1.22	0.03	0.94	0.95
Scalar	110.68	82	0.02	1.35	0.04	0.91	0.91

The metric invariance explains whether girls and boys answered to the items in a similar way. The obtained data for metric factor structure of boys or girls (Chakraborty, 2017) fit acceptable with the theoretical factor structure. Except the TLI value, other obtained values crossed the threshold for the rest of the goodness of fit measures (Table 5). Scalar estimates for every item in both groups were significant on the level *p* < 0.001. Based on this results we can conclude that there are no differences between girls and boys in the way how they answered to the items.

The scalar variance compares the means of the construct across gender groups to check if the observed scores and the latent scores are related (Chakraborty, 2017). On this level we found statistically significant differences (*p* = 0.02) and two of the indexes TLI and CFI do not cross requested threshold (Table 5). Those results showed that children with the same latent construct score did not have same observed scores with respect to the sex membership.

The differences between RMSEA values of configural, metric, and scalar levels are equal to 0.01 which is considered as a good indicator of invariance (Rutkowski and Svetina, 2014; Putnick and Bornstein, 2016). Usually the differences in CFI are reported and requested threshold for change is −0.01 (Cheung and Rensvold, 2010). In our study, there are bigger differences, but Rutkowski and Svetina (2014) permitted the difference −0.02, which is fulfilled between configural and metric level. Based on this results, we assumed that HPSQ-C is sex invariant on the configural and metric level, but the stricter level of scalar invariance is probably much less certain.

### Internal Consistency

Firstly, we checked the internal consistency following the theoretical background. We employed the JASP Team (2019) to calculate the overall McDonald’s ω as well as ω of three factors (legibility, performance time, and physical and emotional well-being) in each questionnaire. Based on McDonald’s ω = 0.91 the reliability of overall HPSQ is considered as excellent. First subscale (legibility) had ω = 0.87, the second subscale (performance time) had ω = 0.77, and finally, the last subscale (physical and emotional well-being) had ω = 0.81. The lowest corrected correlation between item and total score was found in item 6, where *r* = 0.49. During the analysis of particular subscales, we did not find any items that should be removed.

In the case of HPSQ-C, the overall reliability is identified as acceptable (ω = 0.70). In this questionnaire the first subscale (legibility) had ω = 0.67, the second subscale (performance time) had ω = 0.46, and the third subscale (physical and emotional well-being) had ω = 0.57. Two corrected item-total correlations with the overall score were <0.3. More specifically, the corrected item-total correlation for item 3 was *r* = 0.27 and for item 6 was *r* = 0.21. Nevertheless, removal of item 3 did not increase the value of McDonald’s ω of the second subscale (performance time). Only the third subscale McDonald’s ω (physical and emotional well-being) increases to 0.59 when eliminating the item 6.

Additionally, the McDonald’s ω was computed in the proposed models from the CFA analysis (we used the semTools package; Jorgensen et al., 2019). McDonald’s ω of HPSQ for factor legibility was 0.87, for factor performance time it was 0.76, and for the last factor physical and emotional well-being it was 0.85. The overall ω for HPSQ was 0.93. McDonald’s ω of HPSQ-C for factor legibility was 0.66, for factor performance time it was 0.46, and for the last factor physical and emotional well-being it was 0.60. The overall ω for HPSQ was 0.74.

### Discriminant Validity

#### Sex Differences

Based on the independent *t*-test, sex differences were observed when assessed by both questionnaires. Leven’s homogeneity tests were non-significant in both questionnaires: HPSQ (*F* = 0.97, *p* = 0.33) and HPSQ-C (*F* = 1.44, *p* = 0.23). Teachers evaluated boys as worse (HPSQ: *m* = 12.53, SD = 6.91, SE = 0.54) than girls (HPSQ: *m* = 10.34, SD = 6.47, SE = 0.56) with 2.2 difference [95% CI (0.64, 3.74)], which is significant [*t*(292) = 2.78, *p* = 0.006] and has medium effect size *d* = 0.33. Similarly, boys perceived themselves as worse (HPSQ-C: *m* = 13.66, SD = 5.90, SE = 0.46) than girls (HPSQ-C: *m* = 11.89, SD = 5.27, SE = 0.46) with 1.77 difference [95% CI (0.48, 3.07)], which is significant as well [*t*(292) = 2.69, *p* = 0.008] and has medium-sized effect *d* = 0.32.

#### Relationship to Grades

Using Pearson’s correlation coefficients, we observed significant correlation between average grade and total score of HPSQ (*r* = 0.54, *p* < 0.01) and slightly weaker but still significant correlation with total score of HPSQ-C (*r* = 0.28, *p* < 0.01).

### Differences Between Questionnaires

On average, children perceived themselves more strictly (*m* = 12.86; SE = 5.68) than teachers did (*m* = 11.55, SE = 6.79). This difference, 1.32, CI (0.30, 2.33) was significant *t*(294) = 2.55, *p* = 0.011 and it is represented by a small effect size *d* = 0.21.

Pearson’s correlation coefficients were computed to assess the relationships between the items of both questionnaires. Table 6 summarizes all correlation coefficients and related *p*-values and the correlation between the same items of both questionnaires are highlighted in bold. Items 7, 8 (physical and emotional well-being), and 9 (performance time) did not correlate significantly. The highest correlation is between the items with the number 1 (legibility; *r* = 0.34, *p* < 0.01) and 3 (performance time; *r* = 0.32, *p* < 0.01) of both questionnaires. There was a weak positive correlation between the total scores of HPSQ and HPSQ-C (*r* = 0.37, *p* < 0.01).

**TABLE 6:** Pearson’s correlation coefficients between the items of HPSQ and HPSQ-C.

		HPSQ-C											HPSQ										
		1	2	3	4	5	6	7	8	9	10	Sum	1	2	3	4	5	6	7	8	9	10	Sum
HPSQ-C	1	1																					
	2	0.433^∗∗^	1																				
	3	0.180^∗∗^	0.128^∗^	1																			
	4	0.149^∗^	0.141^∗^	0.211^∗∗^	1																		
	5	0.227^∗∗^	0.234^∗∗^	0.098	0.213^∗∗^	1																	
	6	0.146^∗^	0.096	0.193^∗∗^	0.070	0.111	1																
	7	0.202^∗∗^	0.110	0.084	0.176^∗∗^	0.177^∗∗^	0.167^∗∗^	1															
	8	0.196^∗∗^	0.128^∗^	0.195^∗∗^	0.312^∗∗^	0.399^∗∗^	0.189^∗∗^	0.310^∗∗^	1														
	9	0.188^∗∗^	0.194^∗∗^	0.148^∗^	0.270^∗∗^	0.127^∗^	0.016	0.191^∗∗^	0.223^∗∗^	1													
	10	0.417^∗∗^	0.333^∗∗^	0.115^∗^	0.159^∗∗^	0.189^∗∗^	0.049	0.138^∗^	0.185^∗∗^	0.166^∗∗^	1												
	Sum	0.580^∗∗^	0.516^∗∗^	0.434^∗∗^	0.529^∗∗^	0.578^∗∗^	0.321^∗∗^	0.507^∗∗^	0.648^∗∗^	0.501^∗∗^	0.531^∗∗^	1											

HPSQ	1	**0.336**^∗∗^	0.289^∗∗^	0.104	0.204^∗∗^	0.156^∗∗^	0.139^∗^	0.040	0.188^∗∗^	0.108	0.202^∗∗^	0.330^∗∗^	1										
	2	0.290^∗∗^	**0.279**^∗∗^	0.164^∗∗^	0.226^∗∗^	0.090	0.173^∗∗^	0.009	0.145^∗^	0.127^∗^	0.200^∗∗^	0.310^∗∗^	0.805^∗∗^	1									
	3	0.186^∗∗^	0.223^∗∗^	**0.332**^∗∗^	0.260^∗∗^	0.139^∗^	0.194^∗∗^	–0.009	0.152^∗∗^	0.095	0.169^∗∗^	0.319^∗∗^	0.528^∗∗^	0.621^∗∗^	1								
	4	0.261^∗∗^	0.310^∗∗^	0.150^∗∗^	**0.261**^∗∗^	0.103	0.121^∗^	0.015	0.153^∗∗^	0.111	0.205^∗∗^	0.313^∗∗^	0.594^∗∗^	0.562^∗∗^	0.542^∗∗^	1							
	5	0.184^∗∗^	0.177^∗∗^	0.198^∗∗^	0.199^∗∗^	**0.175**^∗∗^	0.125^∗^	0.002	0.169^∗∗^	0.087	0.121^∗^	0.271^∗∗^	0.558^∗∗^	0.602^∗∗^	0.559^∗∗^	0.620^∗∗^	1						
	6	0.170^∗∗^	0.219^∗∗^	0.187^∗∗^	0.158^∗∗^	0.027	**0.199**^∗∗^	0.015	0.097	0.047	0.097	0.213^∗∗^	0.413^∗∗^	0.450^∗∗^	0.363^∗∗^	0.343^∗∗^	0.492^∗∗^	1					
	7	0.079	0.181^∗∗^	0.221^∗∗^	0.190^∗∗^	0.149^∗^	0.120^∗^	**0.090**	0.191^∗∗^	0.058	0.084	0.260^∗∗^	0.387^∗∗^	0.400^∗∗^	0.470^∗∗^	0.452^∗∗^	0.510^∗∗^	0.186^∗∗^	1				
	8	0.137^∗^	0.175^∗∗^	0.263^∗∗^	0.254^∗∗^	0.153^∗∗^	0.090	–0.068	**0.114**	0.102	0.108	0.249^∗∗^	0.527^∗∗^	0.608^∗∗^	0.645^∗∗^	0.611^∗∗^	0.787^∗∗^	0.426^∗∗^	0.519^∗∗^	1			
	9	0.099	0.116^∗^	0.205^∗∗^	0.268^∗∗^	0.070	0.111	–0.045	0.065	**0.056**	0.119^∗^	0.194^∗∗^	0.381^∗∗^	0.422^∗∗^	0.558^∗∗^	0.489^∗∗^	0.474^∗∗^	0.260^∗∗^	0.225^∗∗^	0.534^∗∗^	1		
	10	0.294^∗∗^	0.238^∗∗^	0.178^∗∗^	0.191^∗∗^	0.191^∗∗^	0.150^∗∗^	0.007	0.162^∗∗^	0.076	**0.230**^∗∗^	0.321^∗∗^	0.625^∗∗^	0.626^∗∗^	0.440^∗∗^	0.572^∗∗^	0.566^∗∗^	0.389^∗∗^	0.374^∗∗^	0.537^∗∗^	0.325^∗∗^	1	
	Sum	0.271^∗∗^	0.289^∗∗^	0.278^∗∗^	0.304^∗∗^	0.176^∗∗^	0.187^∗∗^	–0.002	0.186^∗∗^	0.119^∗^	0.205^∗∗^	**0.373**^∗∗^	0.775^∗∗^	0.815^∗∗^	0.787^∗∗^	0.782^∗∗^	0.835^∗∗^	0.562^∗∗^	0.589^∗∗^	0.843^∗∗^	0.659^∗∗^	0.729^∗∗^	1

*^∗∗^Correlation is significant at the 0.01 level (two-tailed). ^∗^Correlation is significant at the 0.05 level (two-tailed).*

## Discussion

The primary purpose of this study was to adapt and evaluate the psychometric qualities of HPSQ and HPSQ-C as screening tools among children in the Czechia. The secondary purpose was to compare both questionnaires because there is no information about their psychometric qualities in one context. The questionnaires were designed as screening tools for identification of handwriting difficulties in children population. We replicated Rosenblum’s (2008) studies (Rosenblum and Gafni-Lachter, 2015) and validated her screening questionnaires in a Czech cohort. As mentioned before, in the Czechia, there is no standardized assessment for D, nor a screening questionnaire, that would enable complex D examination. Moreover, there is no study which compares the psychometric properties of both questionnaires simultaneously.

Initially, both questionnaires were designed as follows: items 1, 2, and 10 should be grouped in factor legibility; items 3, 4, and 9 belong to factor called performance time; and finally, items 5, 7, 6, and 8 are part of physical and emotional well-being factor (Rosenblum, 2008; Rosenblum and Gafni-Lachter, 2015). For the CFA, we built the models based on the theoretical background for each questionnaire separately. The CFA showed poor model fit for the teachers’ model (HPSQ) and excellent model fit for children’s model (HPSQ-C). Correlation between latent factors showed that teachers had a tendency to evaluate children without discriminating based on the theorized factors. In contrast, children perceived those factors as relatively independent. In addition, we checked measurement properties varied by sex, which came out as not significant. We also conduct the invariance analysis for groups of girls and boys with results that suggest possibility of claiming configural and metric invariance for the HPSQ-C, but not scalar invariance.

Previous studies used exploratory factor analysis with different outcomes. Rosenblum (2008) reports two factors in HPSQ. The first factor includes items 3–9 and the second factor comprises items 1, 2, and 10. Her results were confirmed by a Spanish sample (Cantero-Téllez et al., 2015) with the same factor arrangement. Also, in this case, item 6 had the lowest factor score (0.18). Similarly, the original study of HPSQ-C mentions only two factors. The first factor includes item 3 and items from 5 to 9, and the second factor was formed by items 1, 2, 4, and 10 (Rosenblum and Gafni-Lachter, 2015). Based on our results we can conclude that our data for HPSQ did not support the theoretical structure. In contrast, our data support the proposed theoretical structure for HPSQ-C.

We think that the differences between our study and those published in the original study could be caused by the reversal of some items meaning. The same issue with the double negation in the HPSQ items was identified by Schwellnus et al. (2012). Another explanation could be based on the different number of evaluators in both questionnaires. Even when the number of evaluations was the same (*N* = 294), there was a difference in the numbers of independent evaluations of HPSQ (*N* = 21) and HPSQ-C (*N* = 294). Hammerschmidt and Sudsawad (2004) reported that the most important criterion which teachers used for evaluating handwriting issues is legibility (67.8%; *N* = 314). Furthermore, as a major method for handwriting evaluation, they compare student’s handwriting to classroom peers (36.8%). These outcomes support our results. We understand that as an explanation of higher correlations between latent variables in HPSQ model. That is, teachers are probably better in the evaluation of the whole group than of individuals.

Values of McDonald’s ω of the theoretical model indicate excellent (HPSQ, ω = 0.91) and acceptable (HPSQ-C, ω = 0.70) reliability of both questionnaires. Further analysis suggests deleting two items: items 3 and 6 from HPSQ-C. Nevertheless, without item 3, the reliability will not decrease. The total values of McDonald s ω in the proposed CFA model of HPSQ and HPSQ-C could be considered as nearly excellent (ω = 0.93 and ω = 0.74). Both values meet the condition of acceptable reliability for research purposes.

We have a common finding in all results points at item 6 (doing homework). The Sk value 3.34 and the Ku value 12.91 in HPSQ-C indicate that there are very few children who do not do homework. This item was also the less assessed one by both groups, children (*m* = 0.24) and teachers (*m* = 0.39), which again explains minimal problems with homework. We assume that this particular item has minimal discrimination information because almost every Czech child in 3rd and 4th grade do his/her homework. In addition, thanks to the sex invariance analysis in CFA, we know that this issue is related only to the group of girls, because the standardized factor loading of item 6 in HPSQ-C was not significant (*p* = 0.79). This means that this item does not detect differences among girls.

Reliability analysis of HPSQ-C also excludes item 3 (assessing whether a child has enough time to copy text from blackboard). Rosenblum and Gafni-Lachter (2015) wrote about the content validation process. They asked 10 children to complete HPSQ-C questionnaire and rate its items based on their clarity. Each item obtained 100% agreement, nevertheless, two children had a problem with item 3. They reported problems with meaning “repeatedly” in comparison with their classmates. Also, a few teachers from our study had the same difficulties. They complained that the question is not clear. According to them, the time of copying the text from blackboard depends on the length and complexity of sentence or paragraph. Even when internal consistency recommended to delete items 3, 6, and 9, we did not do this. Those items could be more efficient in other age cohorts and further analysis is needed. Moreover, the content of all items is meaningful considering the scope of the questionnaires.

Boys were assessed as worse writers than girls by both groups – teachers (HPSQ) and children (HPSQ-C). These gender differences are a well-known fact, which corresponds with previous research (Blöte and Hamstra-Bletz, 1991; Hawke et al., 2009; Shih et al., 2018). Next, we found out that grades positively correlate with scores of both questionnaires. In addition, worse school achievement is linked with worse handwriting. Similar findings could be found in other studies (Graham et al., 2000; Klein and Taub, 2005).

We found significant differences between total scores made by children (HPSQ-C) and their teachers (HPSQ). Children from our study were more critical during self-evaluation. Similar conclusions are reported by Rosenblum and Gafni-Lachter (2015). The authors write that: “.*children as a whole evaluated their handwriting as less proficient than did their teachers*.” (p. 5). According to outcomes of correlation analysis, there were three items of HPSQ and HPSQ-C which did not correlate: item 7 focused on child’s complaining to pain during the writing, item 8 aimed at fatigue during writing (both from physical and emotional well-being factor) and item 9 surveyed frequency of looking at a blackboard during copying (from performance time factor).

In accordance with the first research studies (Rosenblum, 2008; Rosenblum and Gafni-Lachter, 2015), we found out that children can better distinguish between questions in HPSQ-C questionnaire. Some studies indicate potential disparities between children and teachers/parents in the way of evaluation (e.g., Sturgess and Ziviani, 1996; Bouman et al., 1999; Petersson et al., 2013). Other studies report that children could be better judges of their performance than their parents or teachers (Petersson et al., 2013). Hammerschmidt and Sudsawad (2004) reported that teachers’ evaluation of handwriting problems is not congruent with standardized tests (Sudsawad et al., 2001).

When adults assess children, their opinion could be influenced by their point of view. They are trying to figure out how the child should feel in a situation (Begley, 2000). Teachers cannot discriminate between items and understand them as well as children do. This finding is consistent with that of Germano et al. (2016) who reported that HPSQ did not distinguish dyslectic students. They stated that: “…*teachers perhaps do not have enough knowledge about the handwriting skills*.” (p. 593) as an explanation for higher “never” and “rarely” answer frequency. Based on our results, we can infer that children’s perception of their handwriting is quite different and more accurate.

This study has several limitations. First, we did not control the IQ variable. Nevertheless, the cohort was enrolled in elementary schools where children with mental retardation are usually not included. For that reason, we do not assume this limitation would have a significant impact on our results. Next, we did not compare the results with a baseline diagnosis of D; however, the diagnostic assessment of D in the Czechia is rather subjective and there are problems with establishing the correct diagnosis. On the other hand, the percentage of children with handwriting problems confirmed the estimated prevalence of D in foreign studies (Cermak and Bissell, 2014; Döhla and Heim, 2016). Another limitation could be the different number of independent evaluations collected in each questionnaire as mentioned above.

Another limitation is linked with the teachers’ sample. Variables such are sex, age, or years of experience were not recorded for teachers, therefore they could not be controlled. In future research, especially for the HPSQ these variables should be part of the questionnaire to observe their potential influence on the results. Also, the sample of children consisted only of those enrolled from the 3rd and 4th grades, and this could be seen as non-representative. We chose this range for several reasons: (1) during the 3rd and 4th grades handwriting becomes automatic, (2) thus handwriting issues are more conspicuous and (3) the disorder of written expression (F81.81 in ICD-10) is diagnosed between the 3rd and 4th grades. Nevertheless, Rosenblum and Gafni-Lachter (2015) used HPSQ-C for younger children, i.e., from first grade. Further research in this field is needed.

The last limitation which we want to emphasize is the translation process. The original questionnaire was in Hebrew, but for forward and backward translation we used the English version. Moreover, we did not conduct any of recommended final steps (i.e., cognitive interview, expert panel, or pilot study). Given that the items of the questionnaires are quite simple in terms of comprehension, we did not assume any difficulty of understanding from the children’s or teachers’ points of view. This statement is supported by the fact that there were no conceptual or terminology discrepancies between versions, nor in the forward or in the backward phases of translation.

In the Czechia, there is no quick and reliable screening tool for D assessment which could help teachers, children, and their parents to recognize handwriting issues. Therefore, in the frame of this study, we adapted HPSQ and HPSQ-C questionnaires for the Czech population and evaluated their reliability and validity. Based on a statistical analysis we recommend the HPSQ-C questionnaire for further research use in the Czech population. We would like to emphasize that this questionnaire is only considered as an auxiliary screening tool which should help teachers to identify children with handwriting difficulties. But some additional and accurate diagnosis is still necessary. To sum up, based on the results we suggest that: (1) a child is a better evaluator of her/his issues and they should be seen through her/his eyes; (2) in the case of further research in other languages, inversion of items 1–3, 6, and 10 should be considered. To develop a full picture of psychometric characteristics of this questionnaire additional studies are needed.

As the next logical step in this research, we are going to extend the D assessment methodology based on HPSQ-C by applying a quantitative analysis of digitized handwriting/drawing and utilization of machine learning. This approach can enable us to identify underlying patterns and processes in children with graphomotor disabilities, which can make the general diagnosis more objective and accurate.

## Data Availability Statement

The datasets generated for this study are available on request to the corresponding author.

## Ethics Statement

The studies involving human participants were reviewed and approved by the Ethical Board of the Department of Psychology from Masaryk University. Written informed consent to participate in this study was provided by the participants’ legal guardian/next of kin and by themselves.

## Author Contributions

SR is the author of both questionnaires, and with KS and JM contributed conception and design of the study. PF, BC, and BL collected the data and organized database. KS and TU performed the statistical analysis and interpretation of data. KS translated both questionnaires and wrote the first draft of the manuscript. JM, VZ, ZG, TU, PF, BC, and BL wrote sections of the manuscript. JH contributed with translation process and language correction of both questionnaires. ZS, JH, and SR provide revision of whole article critically for important intellectual content. All authors contributed to manuscript revision, and read and approved the submitted version of the manuscript.

## Conflict of Interest

The authors declare that the research was conducted in the absence of any commercial or financial relationships that could be construed as a potential conflict of interest.

## Footnotes

1 https://www.apa.org/ethics/code/
